# The biobank for the molecular classification of kidney disease: research translation and precision medicine in nephrology

**DOI:** 10.1186/s12882-017-0669-4

**Published:** 2017-07-26

**Authors:** Daniel A. Muruve, Michelle C. Mann, Kevin Chapman, Josee F. Wong, Pietro Ravani, Stacey A. Page, Hallgrimur Benediktsson

**Affiliations:** 1 Department of Medicine, Snyder Institute for Chronic Diseases, University of Calgary, 3280 Hospital Dr. NW, Calgary, AB T2N 4N1 Canada; 2 Snyder Institute for Chronic Diseases, University of Calgary, Calgary, AB Canada; 3 Department of Medicine, Libin Cardiovascular Institute, University of Calgary, Calgary, AB Canada; 4 Department of Community Health Sciences, University of Calgary, Calgary, AB Canada; 5 Department of Pathology and Laboratory Medicine, University of Calgary and Calgary Laboratory Services, Calgary, AB Canada

**Keywords:** Biobank, Kidney disease, Renal pathology, Database, Precision medicine

## Abstract

**Background:**

Advances in technology and the ability to interrogate disease pathogenesis using systems biology approaches are exploding. As exemplified by the substantial progress in the personalized diagnosis and treatment of cancer, the application of systems biology to enable precision medicine in other disciplines such as Nephrology is well underway. Infrastructure that permits the integration of clinical data, patient biospecimens and advanced technologies is required for institutions to contribute to, and benefit from research in molecular disease classification and to devise specific and patient-oriented treatments.

**Methods and results:**

We describe the establishment of the Biobank for the Molecular Classification of Kidney Disease (BMCKD) at the University of Calgary, Alberta, Canada. The BMCKD consists of a fully equipped wet laboratory, an information technology infrastructure, and a formal operational, ethical and legal framework for banking human biospecimens and storing clinical data. The BMCKD first consolidated a large retrospective cohort of kidney biopsy specimens to create a population-based renal pathology database and tissue inventory of glomerular and other kidney diseases. The BMCKD will continue to prospectively bank all kidney biopsies performed in Southern Alberta. The BMCKD is equipped to perform molecular, clinical and epidemiologic studies in renal pathology. The BMCKD also developed formal biobanking procedures for human specimens such as blood, urine and nucleic acids collected for basic and clinical research studies or for advanced diagnostic technologies in clinical care. The BMCKD is guided by standard operating procedures, an ethics framework and legal agreements with stakeholders that include researchers, data custodians and patients. The design and structure of the BMCKD permits its inclusion in a wide variety of research and clinical activities.

**Conclusion:**

The BMCKD is a core multidisciplinary facility that will bridge basic and clinical research and integrate precision medicine into renal pathology and nephrology.

## Introduction

Acute and chronic kidney diseases encompass a vast range of etiologies that affect patients in all demographics. Kidney disease is associated with significant morbidity and mortality. For example, chronic kidney disease (CKD) is an important risk factor for all-cause and cardiovascular mortality [1]. It is estimated that approximately 3 million Canadians are living with CKD due to the increasing prevalence of type 2 diabetes mellitus, obesity, and hypertension [2–4]. CKD is often caused by an initial insult or disease within the kidney tissue, which causes permanent disruption of normal function and persists into a chronic, progressive condition [5]. To date, the majority of kidney diseases are diagnosed on morphological or clinical grounds with only general biomarkers available to stratify risk, guide care, and monitor therapy. Furthermore, due to an inadequate understanding of disease pathogenesis and a paucity of specific biomarker developments, treatment options are generally non-specific (e.g., use of steroids or anti-proliferative agents) and applied broadly with limited success [2, 6, 7].

There is a great need to better understand the underlying etiology of various kidney diseases. Moreover, the application of genetic and molecular knowledge to the human condition is essential to further incorporate mechanistic data to existing morphologic disease classification. These two fundamental events are essential not only to reveal an individuals’ risk or prognosis with kidney disease, but to also identify improved and targeted treatment options. In combination with well-established clinical parameters and risk models, the incorporation of genetic/molecular data into patient care is, in essence, the definition of precision (or personalized) medicine that will surmount to a substantial shift in the very nature of renal pathology and nephrology practice.

The past decade has been marked by an increase in strategies to bring precision medicine to the forefront of healthcare [6]. As a result, the need to establish appropriate infrastructure to link patients and high quality clinical data to advanced molecular and genetic technologies has become apparent. A cornerstone to achieving this capacity is the development of biobanks that can collect and maintain collections of human biospecimens needed to support precision medicine advances in kidney disease [8–10]. Due to the rising prevalence of kidney diseases and CKD in Canada, as well as the critical need for improved diagnostic testing methods and treatment options in this patient population, in 2015 the University of Calgary established the Biobank for the Molecular Classification of Kidney Disease (BMCKD). In this paper, we describe the planning, execution and operational aspects of the BMCKD. We also review some of the short and long term challenges encountered in the process to serve as a reference for other centres planning to enter into this exciting area of medicine.

## Objectives

The BMCKD is housed within the University of Calgary’s Cumming School of Medicine in Calgary, Alberta and represents the first comprehensive kidney disease biobank in Canada. The BMCKD’s long term objective is to bridge basic science and clinical silos to facilitate the integration of clinical, epidemiological, pathological, molecular and genetic knowledge in kidney disease research and clinical practice. The refinement of kidney disease diagnosis, classification and risk-stratification will enable precise treatments for patients, with the objective to improve health outcomes.

In addition to the long term vision for Precision Medicine in Nephrology, the BMCKD is guided by four principal operational objectives: 1) To efficiently organize and manage retrospective and prospective human renal pathology specimens and data. 2) To establish a population based cohort for renal pathology and glomerulonephritis research. 3) To provide infrastructure for kidney disease research and precision medicine in nephrology that employs human biospecimens. 4) To enhance medical education in renal pathology and glomerulonephritis.

## History and realization of the BMCKD

Renal pathology in Southern Alberta has historically been centralized at the Foothills Medical Centre and managed by the Department of Pathology and Laboratory Medicine (DPLM) at the University of Calgary and Calgary Laboratory Services (CLS), a wholly owned subsidiary of Alberta Health Services (AHS), the province-wide government health care organization. In keeping with the retention guidelines defined by the Canadian Association of Pathology, CLS and the DPLM have accumulated all adult and pediatric kidney biopsies performed in Southern Alberta since the late 1970’s, with some items dating back to the 1960’s. The materials included: 1) paraffin tissue blocks, 2) plastic-embedded tissue blocks for light and electron microscopy, 3) frozen tissue blocks, 4) histopathology slides, 5) detailed pathology reports and 6) electron microscopy images. CLS is the custodian of the aforementioned renal pathology materials. With increasing space demands within CLS, the long-term accumulation of kidney biopsy materials and biospecimens provided an ideal opportunity to leverage this resource into a functional biobank that would serve the needs of multiple stakeholders.

In 2012, a plan to consolidate and reorganize the renal pathology resource in Calgary was created that represented “value added” to the current storage situation and long term sustainability. The BMCKD’s overall mission was to provide an efficient and cost-effective mechanism to deliver renal pathology services for clinical care and to foster kidney disease research employing human biospecimens. The BMCKD plan identified several stakeholders including CLS, The University of Calgary Cumming School of Medicine, The Snyder Institute for Chronic Diseases, Clinical Departments (Medicine, Nephrology, Transplantation, Pathology and Laboratory Medicine, Pediatrics) and patients themselves.

The BMCKD operational plan was approved by the various partners and the project was financed primarily through an infrastructure grant from the Canada Foundation for Innovation (CFI) and investments from the stakeholders. Implementation of the BMCKD plan began in 2013 and the biobank became operational on January 1, 2015.

### Challenges

One of the major hurdles to obtaining an approved operational plan was aligning expectations between research and clinical stakeholders. In particular, entering into a formal agreement with the custodian CLS was essential. Since CLS’ primary mandate is patient care, several months of negotiations were required before an agreement in principle was achieved to release clinical samples and data to the biobank. Conditions around privacy, data security, finances, tissue integrity, and human resources had to remain consistent with CLS rules and regulations. The University of Calgary on the other hand, has a mission centred on innovation, and thus an efficient mechanism to conduct research was also required. Ultimately, the needs of all parties were met by designating the BMCKD as a joint CLS/University of Calgary project where CLS would release de-identified samples and data to the biobank for research, but would remain the custodian of those materials. In return for research access to the materials, the BMCKD would manage renal pathology inventory, data and research protocols, as well as support clinical services according to CLS guidelines. The improvements in efficiency, space requirements and cost, as well as the BMCKD’s proposal to offset some operational expenses through research funding provided CLS with a significant value incentive to participate in the project. Furthermore, as a pilot project for digital pathology services, the BMCKD represented an enticing opportunity for innovation. Patient biospecimens collected as part of University-sponsored research projects would remain outside the scope of the CLS agreement fulfilling an important requirement on the academic side. All of the above conditions were included in a Memorandum of Understanding between the BMCKD, the University of Calgary and CLS that was finalized more than 2 years after the project was initially conceived.

## Infrastructure

**Fig. 1:**
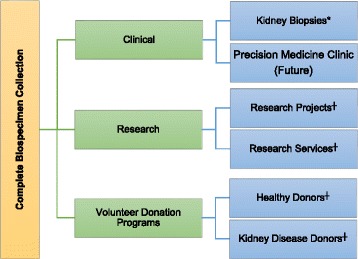
Biospecimen Collection Provision. * contributed to the Biobank under a Memorandum of Understanding with Calgary, Lab Services (custodian),^┼^ contributed to the Biobank under approved ethics certificates, informed consent, research agreements, and non-disclosure agreements

The BMCKD is housed in a 550 ft^2^ wet laboratory space at the University of Calgary, Snyder Institute for Chronic Diseases that allows for both clinical and research use of the collected materials. The BMCKD contains regular shelving and storage containers for paraffin and plastic-embedded tissue blocks. Three −80° freezers with carbon dioxide backup are used for the storage of frozen sections and other human biospecimens. The −80 freezers are equipped with internal racks and shelving that supports standard freezer boxes and the Micronic box and lid storage system (Micronic Inc., Lelystad, The Netherlands). For other human biospecimens, the BMCKD employs both 1.4 ml and 6.0 ml etched bar-code Micronic tubes and storage boxes. Inventory is tracked using 1D and 2D barcode scanning equipment and software. Biospecimens are collected through a variety of channels which are discussed further below (Fig. 1).

The BMCKD also is equipped to perform molecular pathology research. Microtomes and associated equipment allow cutting and staining (standard and immunochemistry) of frozen, paraffin and plastic tissue sections. Slides are imaged using wide-field fluorescence microscopy available in the BMCKD or within the adjacent Snyder Institute Live Cell Imaging Facility that contains confocal microscopy and other state-of-the-art imaging equipment. Additionally, the BMCKD is equipped to perform real time PCR as well as DNA/RNA/protein imaging and quantification. Basic science laboratories in the Snyder Institute for Chronic Diseases support the BMCKD infrastructure by providing biosafety cabinets, centrifuges and freezer monitoring equipment.

### Challenges

Planning the BMCKD infrastructure required consideration of the budget and existing institutional resources. Since it was not fiscally feasible to perform all possible biobanking procedures, it was decided to focus the BMCKD on 2 core technical competencies a) provide core biobanking support for CLS and the research community, including a minimum capacity to store human biospecimens for a minimum of 10 years, and b) have the capability to perform molecular pathology and immunohistochemistry on kidney biopsies. In this manner, the BMCKD would maximize its effectiveness and provide non-redundant infrastructure to the local research environment. Maintaining realistic operational objectives remains key to achieving a sustainable core facility.

Several other minor considerations included the choice of specimen containers, inventory and bar-coding equipment which needed to account for a wide range of potential applications and ensure future compatibility. The decision to use the Micronic platform was due to its wide range of tube sizes, screw-top lids, barcode durability, and inventory capability for both the tubes and racks. The storage of frozen kidney tissue blocks is also problematic. Frozen kidney tissues are embedded in Optimum Cutting Temperature (OCT) compound and stored in standard freezer boxes at −80°. One major issue with this storage method is tissue dehydration. Storage of frozen tissue sections in liquid nitrogen may improve long term tissue integrity but is associated with significant cost. Finally, one must consider ongoing maintenance and service contracts for all specialized equipment.

## Information technology infrastructure

One of the central aspects of the BMCKD is an information technology infrastructure with cross-referencing capability. The biobank consists of 3 separate databases hosted on virtual machine servers in a secure environment on the University of Calgary network. The secure environment was designed to store patient-identifying information with high level data privacy and security provisions that would ensure compliance with the Health Information Act of Alberta [11]. User access on all platforms is restricted with multiple levels of credentials. Database access and use is monitored and audited on a frequent basis.

The first database is built on the REDCap (Research Electronic Data Capture) platform which is a secure, web-based application designed to support data capture and export procedures for research studies [12]. The BMCKD REDCap database contains the renal pathology reports associated with every kidney biopsy performed in Southern Alberta since 1962. The data dictionary corresponds to the current fields of clinical reports (Table 1). The variables will be expanded and refined as information is parsed and the database matures over time. As of June 2017 the database contains 14,027 kidney pathology reports from 9988 patients (Table 2). The database is completely de-identified by the custodian (CLS), but contains a unique and coded patient number. CLS holds the master key to these patient codes, which can be made available to the BMCKD only in the event that patient identifying information is required and following approval for research purposes.

**Table 1:** REDCap Patient Database Variables

Database	Data Variable
Patient	Database Record ID number
	Unique CLS Patient ID number
	Consent (Y/N)
	Patient birth year
	Patient birth month
	Patient Sex (M/F)
Encounter (Biopsy)	Unique CLS Biopsy number (Surgical accession number)
	Biopsy Date
	Specimen
	Specimen Description
	Transplant (Y/N)
	Diagnosis
	Gross Description
	Microscopic Description
	Comment

**Table 2:** REDCap Kidney Biopsy Data Summary

Variable	Number (n)
Patients	9988
Sex	
Male	5679
Female	4309
Biopsies	14,027
Type	
Transplant	4307
Native	9720

The second database uses the Freezerworks platform (Dataworks Development Inc., Mountlake Terrace, WA, USA) and holds data regarding the biospecimen inventory. Kidney tissue blocks as well as biospecimens (blood, urine, DNA, RNA, etc.) associated with specific research projects are held within this database. Kidney biopsy specimens (frozen, paraffin and plastic) are de-identified by CLS and organized according to the same coded patient number as in REDCap allowing for cross-referencing of pathology reports and tissue. The database monitors tissue usage, sectioning and handling (deposit and retrieval) by research and clinical personnel. Freezerworks will have a multi-user capacity and log biospecimens for specific research studies. In these cases, a separate database within the program will be created for each research project. Since the data held within these databases may contain patient identifying and other health information depending on the specific protocol, Freezerworks is hosted in a secure environment on the University of Calgary network with a Privacy Impact Assessment in place as mandated by section 64 of the Health Information Act of Alberta [11] and approved by the Office of the Information and Privacy Commissioner in the Province of Alberta (www.oipc.ab.ca). These projects may fall outside of the renal pathology domain of the BMCKD and may not necessarily contain the same unique patient number derived by CLS. For research initiatives, cross-referencing and linking samples to identifiable health information is possible, contingent on research ethics certification.

The third database consists of the renal pathology digital image repository. This database is being populated with images created by an Aperio Scanscope AT2 instrument using the eSlide Manager software (Leica, Wetzlar, Germany). To date the BMCKD has digitalized 10,333 cases which includes 104,527 renal pathology slides and electron microscopy images to create a digital resource for clinical use, research and education. The digital image database consists of all renal pathology cases since 1990 and comprise close to 75% of all kidney biopsies ever performed in Southern Alberta. Cases prior to 1990 will only be digitalized on request for clinical or research purposes. Prospective scanning of renal pathology is ongoing. The eSlide Manager database is also organized using the coded patient number derived by CLS. The database is being utilized for digital renal pathology clinical services. Thus patient identifiers have been maintained within the database to ensure data integrity and patient safety. This information is protected and only accessible by CLS authorized staff. Again, since this database contains patient identifying health information that may be used for clinical as well as research purposes, a Privacy Impact Assessment is in process as per the Health Information Act of Alberta [11].

### Challenges

The major challenge in establishing the IT infrastructure revolves around ensuring adequate data security and compliance with existing privacy legislation. A thorough understanding of institutional guidelines and legal aspects of creating patient-level data repositories is essential. In our experience, we consulted frequently with privacy officers within the University and AHS to ensure the biobank was compliant.

Ongoing server maintenance, data management, software upgrades and memory usage are other often overlooked issues. These are continuing costs that must be factored into any biobank plan. Software choices must also consider compatibility issues needed to enable participation in local, national and international studies or networks.

## Operational objectives

The overarching goal of the BMCKD was to create a multi-purpose infrastructure to serve research, clinical and educational mandates. This multidisciplinary approach establishes an integral link between clinical medicine and research as well as a foundation for long-term sustainability. The BMCKD now prospectively manages kidney biopsies and associated data to act as the primary resource for all kidney disease research and clinical care that involves renal pathology or human biospecimens. As stated above, the operational objectives must remain focused and fiscally feasible in order for the biobank to be effective. The objectives of the BMCKD are:

***1) Efficiently organize and manage retrospective and prospective human renal pathology specimens and data*** *.* Prior to the BMCKD plan, renal pathology was organized through the physical storage of tissue blocks, slides and electron micrographs at multiple sites. Inventory of renal pathology specimens and data was rudimentary and maintained on written registers or simple computer spreadsheets within the clinical laboratory. The organization and retrieval of renal pathology specimens for clinical care or research was laborious and space-intensive due to retention guidelines for human pathology specimens.

All of the retrospective renal pathology specimens and data were transferred to the BMCKD by its custodian CLS for storage and reorganization using the three data platforms as described above. All renal pathology materials within the BMCKD are de-identified with the exception of the digitalized histopathology, where patient identifying information is maintained to ensure data integrity and patient safety, but suppressed to protect patient confidentiality. A unique coded patient number that is common across all BMCKD platforms is managed by CLS and can be released for research under an appropriate ethics certification. With the exception of the tissue blocks, all physical materials are then placed into long term storage at an Iron Mountain storage facility in Calgary.

Following the reorganization of the retrospective cohort, the BMCKD is now populated prospectively with renal pathology materials after the cases have been signed out by the pathologist and completed their primary clinical purpose. Similar procedures used for the retrospective cohort will be maintained. Overall, the BMCKD will become the single resource for renal pathology in Southern Alberta that will improve access for clinical care, research, education, reduce space requirements, and lower costs.

***2) Establish a population based cohort for renal pathology and glomerulonephritis research*** *.* Glomerulonephritis represents a relatively rare group of kidney diseases [13]. As such, significant advance in glomerulonephritis research is hampered by low patient numbers and insufficient power in clinical trials. The centralized accumulation of kidney biopsy material and data by CLS and the DPLM allowed the assembly of a population based cohort of patients with glomerulonephritis and other rare kidney diseases. The secondary research use of all past and present kidney biopsy specimens collected as part of clinical care creates a wide range of renal pathology research opportunities including epidemiological, genetic and molecular biomarker, disease classification and quality assurance/improvement studies. For example, information from the BMCKD could be used to assess the evolution of physician practice, health resource utilization, disease patterns or patient demographics over time. Similarly, the linkage of molecular, genetic and renal pathology data to administrative and clinical databases will provide insights todisease prognosis and outcomes. Collectively these studies could inform health care policy, enable more precise medical practice or generate hypotheses for clinical and basic research. The BMCKD currently has more than 14,000 kidney biopsies and associated data from more than 9500 patients (Table 2). The number of cases will increase further as material is collected prospectively, and should reach 20,000 in the next 10 years based on local annual biopsy rates.

***3) Provide infrastructure for kidney disease research and precision medicine in nephrology that employs human biospecimens*** *.* With the recent expansion in systems biology and precision medicine approaches in nephrology research and clinical practice, the demand for biobanking is increasing [7–10, 14]. The BMCKD infrastructure will provide critical support for translational research and precision medicine in nephrology through its capability to process and store patient biospecimens that includes kidney tissue, cells, blood, urine, DNA, RNA and protein. These activities will enable interrogation of molecular and genetic pathways of human disease using advanced technologies and will encourage the participation and conduct of clinical and basic science research that employs human biospecimens. The biobank includes a healthy volunteer program to establish a pool of control biospecimens for research. A process in which participants may consent to contributing biospecimens such as blood, urine, DNA or tissue (nephrectomy samples) to the biobank is established. In the absence of such infrastructure, these studies are more difficult to perform and significantly more expensive. A clear path for acquiring, storing and analyzing human biospecimens is invaluable for encouraging and enabling translation studies and precision medicine in kidney disease. It is also essential to access national or international networks, consortiums or clinical trials.

***4) Enhance medical education in renal pathology and glomerulonephritis*** *.* The establishment of a digital platform for renal pathology will facilitate education for medical students, residents and fellows as well as continuing medical education in nephrology and pathology. A single clinical database will allow for rapid retrieval of data and images, including the ability to review large cohorts to assess and acquire knowledge pertaining to the variability and complexity of kidney pathology. The digitalization of images will greatly facilitate teaching rounds and coursework.

## Governance

Given the scale of the current and future BMCKD activities, the creation of a governance structure was essential [15]. All clinical and research activities are overseen by the BMCKD’s Management Committee. The Management Committee meets bi-annually to provide general, high-level oversight and input on research and clinical activities involving the biobank, to review and adjudicate incoming research application requests, and to discuss the overall direction of the biobank in regards to future targets and funding opportunities.

**Fig. 2:**
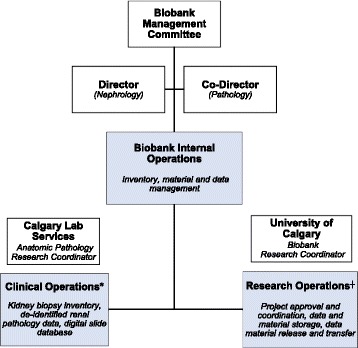
BMCKD governance structure,* conducted under a Memorandum of Understanding with Calgary Lab Services (custodian), ^┼^ conducted under approved ethics certificates, informed consent, research agreements, service agreements, data and material transfer agreements, and non-disclosure agreements

On a smaller scale, all BMCKD activities are led by the appointed Director and Co-director, who oversee all daily internal operations within the biobank and its associated laboratories. The BMCKD’s internal operations are dictated by the needs of the ongoing clinical and research activities, both of which are managed by the designated coordinator (Fig. 2). The composition of the management committee and the duties of the various BMCKD personnel can be found at http://www.snyder.ucalgary.ca/research/precision-medicine-nephrology.

## Standard operating procedures

All clinical and research operational activities are implemented by the BMCKD’s policies and standard operating procedures (SOPs). Each policy and SOP is reviewed on an annual basis by the Research Project Coordinator, who also maintains the SOP index through an iterative process which follows the expansion of the biobank’s activities (Table 3). These procedures are critical to ensure the integrity of the BMCKD sample and data collection. They also serve to facilitate educational opportunities and training for researchers or other individuals involved in some capacity with the biobank.

**Table 3:** Standard Operating Procedure (SOP) index

Procedural Topic	Title
Administration	Obtaining Non-Disclosure Agreements
	Administration of SOPs
	Staff Roles and Responsibilities
Participant Recruitment and Management	Participant Recruitment
	Developing and Revising Consent Forms
	Obtaining Informed Consent
	Withdrawal of Consent
Records Management and Documentation	Information Access Control
	Document Maintenance
Facilities Management and Operations	Physical Security
	Emergency Preparedness
Quality Assurance	Assessing Quality of Tissue Specimens
Safety	Handling Hazardous Chemical Waste
Training	Education and Training
Material Handling and Documentation	Biohazardous Material Waste Management
	Blood Collection
	Urine Collection
	Sample Retrieval and Transfer
	Labeling and Tracking Materials
Material Release	Sample Shipping and Transportation
	Completion of a Material Transfer Agreement
	Material Request and Release
	Return of Biospecimens for Clinical Purposes

## User Interface

The BMCKD will have multiple user interfaces. First, the BMCKD will provide a clinical interface through an ongoing interaction with CLS and the DPLM. Digital renal pathology images are available to pathologists and eventually nephrologists through a web-based platform. This interface heralds the era of digital pathology in Southern Alberta providing access to renal pathology for review. Although initial renal pathology will be performed using standard glass slides and microscopy, the digital image repository will be useful for reviewing historical cases and to provide access (including remote access) for the primary nephrologists as part of longitudinal patient care. Eventually, the BMCKD digital repository could be linked to the Province-wide digital medical record, Alberta Netcare. The BMCKD will also manage kidney biopsy specimens for CLS. Tissue blocks that are required for further staining or analysis will be available and tracked using the Freezerworks program. There will be ongoing dialogue and biospecimen traffic as the BMCKD collects renal pathology specimens and data prospectively.

**Fig. 3:**
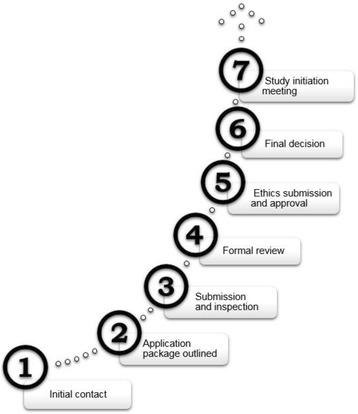
Research project application process flow

Researchers will access materials in the BMCKD through a defined, step-wise research application process (Fig. 3). Investigators will be asked to provide a summary of their protocol, budget and ethics certification prior to review by the Management Committee. Approval of research projects will then occur and investigators will be provided with their specific requests depending on the protocol. Research activities in the BMCKD include the collection, processing, storage and transfer of biospecimens and clinical data for investigators at the University of Calgary and throughout Alberta. Data are extracted from the databases and provided in simple format on a restricted shared drive. No researcher will have direct access to the databases. All projects approved by the Management Committee must also be approved by the University’s ethics board prior to initiation. Projects are provisioned according to the terms and conditions outlined in their respective research agreements, material transfer agreements, and non-disclosure agreements between the BMCKD and Principal Investigator. Research personnel have the potential access to a number of resources to support their projects, including de-identified patient data from past biopsies (CLS data), de-identified data from previous research projects, as well as biospecimens from previous biopsies or research collections.

Similarly, for educational endeavors and rounds, specific requests for material will be made to the Management Committee prior to releasing information in a controlled manner and under appropriate data, non-disclosure and materials transfer agreements.

## Ethical framework

The evolving ethical aspects of creating, maintaining, and utilizing a human biobank for research and clinical purposes are complex [15–19]. While the general nature of collecting and storing personal information in a clinical sense is not new, there are growing concerns regarding the ethical nature of collecting, storing, processing, and genetically profiling human biospecimens and their associated health data for precision medicine applications and future research. Under the Tri-Council Policy Statement 2 (2014) Ethical Conduct for Research Involving Humans [20] and the Health Information Act of Alberta [11] individual level consent is not required for research use of de-identified information, whether this is biospecimens or health information. Where the data are identifiable, different considerations apply. The collection of sensitive data and biospecimens requires a great deal of consideration regarding the ethical, security, and confidential nature of all activities within the biobank [16].

The BMCKD operates under an ethics certification which recognizes that the renal pathology materials, originally collected as part of clinical care, are now stored in a de-identified form and may subsequently be used for research purposes. This ethics certification does not permit blanket use of these materials for research, nor does it allow access to patient identifying (health) information. Thus, the BMCKD functions with a waiver of patient consent for the purpose of storing materials for future secondary use. The next level of BMCKD’s ethical framework is project specific and may include consent considerations as required under the Health Information Act [11]. All individuals who utilize BMCKD resources for research must submit their protocols to the BMCKD Management Committee as well as the University of Calgary Conjoint Health Research Ethics Board (or applicable Ethics committee for external sites) for their respective approvals prior to initiation of the study. Depending on the proposed use of the biobank materials or resources for specific projects, independent evaluation of research protocols submitted by prospective investigators will determine the steps required, including the possible need to obtain individual consents. In the case where consent is required, potential study participants must be informed of the original, clinical sample collection and storage procedures of BMCKD and the proposed subsequent research use(s). Participants must provide written consent to acknowledge their understanding of the proposed research including what the samples will be used for, storage and privacy provisions. At no point in time will any identifiable information collected be made available to any other researcher or individual accessing these biospecimens in the future.

### Challenges

Due to the fact that large, multi-purpose biobanks like the BMCKD are relatively new and therefore largely unregulated, creating an effective ethical framework required a significant amount of planning and consultation with the ethics board. In addition, during the period that the biobank was being operationalized, a number of changes in privacy legislation and thus ethical guidelines occurred that required reassessment and modification of previously approved protocols. Thus, the ethical framework of the BMCKD will require ongoing review and change as precision medicine moves towards the forefront of healthcare. Hopefully as institutions become more familiar with biobanking for clinical and research purposes, regulatory requirements surrounding it will become much more concise and easily adoptable.

## Legal framework

Use of the BMCKD’s renal pathology materials for research and the creation of digital histopathology database for clinical care, is bound by a Memorandum of Understanding between the BMCKD, the University of Calgary and the custodian of the materials CLS. The Memorandum of Understanding outlines the approved terms and conditions of the storage and use of retrospective and prospective clinical biopsy samples and associated materials. Use of the BMCKD materials for clinical care is bound by the Health Information Act of Alberta [11]. As aforementioned, any digital slides, biopsy samples or data released for research purposes must be approved by the Management Committee and the appropriate Ethics Board and are subject to conditions set within the ethics certification and a number of binding contracts between the BMCKD and the researcher.

Issues of legality and ownership of samples in respect to biobanks remain widely debated topics [16, 21, 22]. Due to the fact that large, multi-purpose biobanks like the BMCKD are relatively new, biobanking remains an unregulated issue which is reliant on a number of binding contract and service agreements. Under the privacy and health information laws of the Province of Alberta, the BMCKD is obligated to conceal all identifying and health information of biobank participants at all times. All biobank personnel and affiliated researchers and physicians are required to sign legally binding confidentiality agreements, and safety and security SOPs outline measures in which information privacy is maintained at all times (Table 3). Biospecimens collected through clinical care and submitted to the BMCKD under the provision of the custodian CLS, are protected by de-identification of any personal data and governed by the Health Information Act [11]. Biospecimens, personal or health information acquired by the BMCKD outside of clinical care is guided by specific research protocols and their associated ethics certification.

For research purposes, principal investigators engaging in an approved research project must sign a Research Agreement or Service Agreement which outlines the appropriate use of provided de-identified data or the biospecimen collection services provided by the BMCKD. Researchers requesting access to stored biospecimens or digitalized histopathology images must also sign a Data or Material Transfer Agreement and Non-Disclosure Agreement in order to uphold the highest level of confidentiality and security of provided material. All BMCKD contracts and agreements have been approved by the University Cumming School of Medicine Legal Department.

The most widely contested legal aspect of biobanking pertains to ownership of the collected biospecimens once they are stored [16, 21, 22]. Legal precedent exists for the transfer of ownership of human tissue/biospecimens procured for clinical care to the medical institution where the procedure was performed. Others may argue that the patient shall always remain the owner of the sample, with the ability to retract it and any related data at any time [22]. With regards to renal pathology materials in the BMCKD governed under the existing Memorandum of Understanding, the custodian CLS is responsible for ownership issues under the applicable laws of the Province of Alberta and Canada. For biospecimens collected specifically for research following written, informed consent, the BMCKD will assume guardianship and release biospecimens only for the purposes outlined within research projects having both Ethics and BMCDK Management Committee approval. Each patient will remain the owner of their own samples and therefore maintain the right to request the withdrawal and destruction of all records and biospecimens within the BMCKD.

Given these important issues relevant to patient stakeholders, the BMCKD has a patient representative on the management committee and will be engaging patients and the community at large through a number of patient oriented research programs, such as the Alberta Strategy for Patient Oriented Research (SPOR) Support unit (http://www.aihealthsolutions.ca/initiatives-partnerships/spor).

## Future challenges and sustainability

The establishment of a functioning biobank is the first step in developing a program in precision medicine where patient biospecimens are regularly utilized in research and ultimately clinical care. One of the first challenges is to ensure visibility of the BMCKD program, stakeholder and user buy-in. While the general premise and prospect of biobanking has gained traction among academic clinicians and researchers, operationalization of precision medicine programs remains uncommon. In order for a human specimen repository to be effectively integrated into the core operational infrastructure of an institution, interest must be fostered in prospective partners, researchers, staff, and patients through effective engagement strategies. Inclusion of various department heads, research labs, and clinical programs in the planning, initiation, execution, and expansion of various aspects of the biobank is often an effective method of gaining stakeholder buy-in. Similarly, the inclusion of patients and community members in various research projects and clinical programs associated with the biobank is of critical importance in order to increase visibility and increase the biospecimen collection volume. Advertisements, web pages, social media platforms, and lay press are all excellent ways to reach out to potential researchers as well as study subjects. For example, besides the recruitment of research participants with specific kidney diseases, the BMCKD is creating a community engagement strategy to promote the healthy volunteer donation program which will serve to increase the quantity of healthy blood and urine samples that may be used in projects also requiring normal controls.

Long term, usage and financial stability are the two primary goals to achieving long term sustainability. For any biobank to be effective, it must be used by researchers, clinicians and educators. The BMCKD was formulated around renal pathology processes, and many projects in this domain can be executed locally. The examination of glomerulonephritis and kidney pathology at a population level is a unique capability for the facility. The general ability to provide biobanking support will hopefully attract translational and clinical research studies that incorporate systems biology approaches, which will in turn allow the BMCKD to attract peer-reviewed funding for its intrinsic research operations. The BMCKD directors will actively pursue research collaborations locally, nationally and internationally to ensure the data and materials are incorporated into clinical and basic science research projects further attracting research dollars. Efforts will also be made to encourage quality assurance and quality improvement projects as well as exploiting the resource for educational purposes. Finally, adoption of the BMCKD resources for clinical purposes is probably the most essential and effective mechanism to achieve sustainability. With an effective strategy to improve the quality and efficiency of patient care, support for the BMCKD will hopefully continue from its major health-care stakeholders in CLS and AHS.

## Conclusion

Several kidney-focused biobanks are underway including in the Netherlands and Spain [8, 9]. The BMCKD will be the first comprehensive biospecimen and renal pathology resource for researchers and clinicians wishing to conduct translational research and precision medicine for kidney disease in Canada. In its first few years of operation, the BMCKD has achieved a number of targets which will establish the biobank as a leader and model for additional renal biobanks, translational research and precision medicine advancements within the nephrology landscape.

## Abbreviations

AHS: Alberta Health Services
BMCKD: Biobank for the Molecular Classification of Kidney Disease
CFI: Canada Foundation for Innovation
CKD: Chronic Kidney Disease
CLS: Calgary Lab Services
DPLM: Department of Pathology & Laboratory Medicine
SOP: Standard Operating Procedure
